# Digital Mammography (DM) vs. Dynamic Contrast Enhancement-Magnetic Resonance Imaging (DCE-MRI) in Microcalcifications Assessment: A Radiological–Pathological Comparison

**DOI:** 10.3390/diagnostics14111063

**Published:** 2024-05-21

**Authors:** Federica Cicciarelli, Elisa Guiducci, Francesca Galati, Giuliana Moffa, Paolo Ricci, Federica Pediconi, Veronica Rizzo

**Affiliations:** 1 Department of Radiological, Oncological and Pathological Sciences, Sapienza University of Rome, 00161 Rome, Italy; federica.cicciarelli@uniroma1.it (F.C.); francesca.galati@uniroma1.it (F.G.); giuliana.moffa@uniroma1.it (G.M.); paolo.ricci@uniroma1.it (P.R.); federica.pediconi@uniroma1.it (F.P.); veronica.rizzo@uniroma1.it (V.R.); 2 Unit of Emergency Radiology, Policlinico Umberto I, Sapienza University of Rome, Viale del Policlinico 155, 00161 Rome, Italy

**Keywords:** breast microcalcifications, digital mammography, DCE-MRI, radiologic–pathologic correlation, breast cancer

## Abstract

The aim of this study was to compare the characteristics of breast microcalcification on digital mammography (DM) with the histological and molecular subtypes of breast cancer and to identify the predictive value of DM and dynamic contrast-enhanced magnetic resonance imaging (DCE-MRI) in assessing microcalcifications for radiologic–pathologic correlation. We relied on our prospectively maintained database of suspicious microcalcifications on DM, from which data were retrospectively collected between January 2020 and April 2023. We enrolled 158 patients, all of whom were subjected to biopsy. Additionally, 63 patients underwent breast DCE-MRI. Microcalcifications with a linear branched morphology were correlated with malignancies (*p* < 0.001), among which an association was highlighted between triple negatives (TNs) and segmental distribution (*p* < 0.001). Amorphous calcifications were correlated with atypical ductal hyperplasia (ADH) (*p* = 0.013), coarse heterogeneous (*p* < 0.001), and fine-pleomorphic (*p* = 0.008) with atypical lobular hyperplasia (ALH) and fine pleomorphic (*p* = 0.009) with flat epithelial atypia (FEA). Regarding DCE-MRI, no statistical significance was observed between non-mass lesions and ductal carcinoma in situ (DCIS). Concerning mass lesions, three were identified as DCIS and five as invasive ductal carcinoma (IDC). In conclusion, microcalcifications assessed in DM exhibit promising predictive characteristics concerning breast lesion subtypes, leading to a reduction in diagnostic times and further examination costs, thereby enhancing the clinical management of patients.

## 1. Introduction

Breast cancer (BC) is a heterogeneous disease, characterized by diverse histological and molecular patterns, leading to various therapeutic approaches and prognoses [1]. In this context, the importance of early diagnosis is well acknowledged [2].

Digital mammography (DM) stands out as the most sensitive first-level diagnostic tool for detecting microcalcifications, non-palpable masses, breast asymmetry, and parenchymal distortions [3]. While many calcifications identified in mammograms are typically benign and require no further assessment, certain microcalcifications warrant additional evaluation due to their potential association with malignancies, which may only manifest as microcalcifications in mammograms [4].

The Breast Imaging Reporting and Data System (BI-RADS^®^) aims to provide standardized tools for reporting breast lesions observed in mammograms, ultrasound, and dynamic contrast-enhanced magnetic resonance imaging (DCE-MRI) [3].

In the realm of DM, the BI-RADS^®^ 5th edition lexicon specifically describes suspicious calcification morphologies.

To the best of our knowledge, while the correlation between microcalcifications and BC is extensively documented in the literature [5,6], gaps remain, such as the absence of imaging characterizations linking microcalcifications with B3 lesions, a heterogeneous group of lesions, with a wide range of PPV for malignancy ranging from 9.9 to 35.1% [7,8,9,10,11,12].

Moreover, there is a lack of data regarding the diagnostic accuracy of DCE-MRI for patients with new or worsening microcalcifications. Our understanding of the diagnostic efficacy of DCE-MRI in such cases remains inadequate, indicating the need for further research [13].

Breast DCE-MRI can be particularly valuable in cases of microcalcifications with a low risk of malignancy [14], and it is crucial for surgical planning after percutaneous biopsy [15,16]. It supports assessing factors such as lesion size, multifocality, multicentricity, and bilaterality, which are pivotal for individuals considering breast-conserving surgery [15]. When a DCE-MRI identifies an enhancing lesion corresponding to an area with suspicious microcalcifications seen on mammography, biopsy is typically recommended. DCE-MRI has the potential to decrease unnecessary breast biopsies, especially in cases of microcalcifications with a low risk of malignancy.

**Figure 1:**
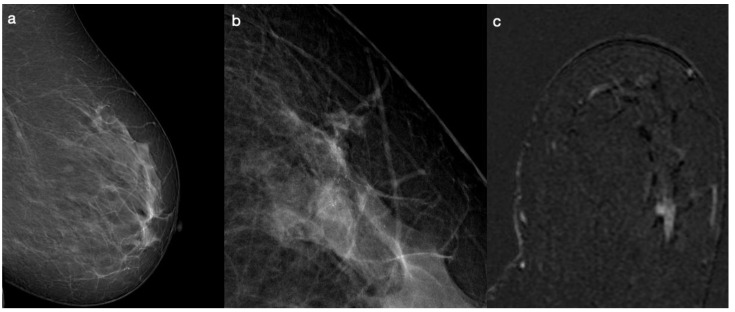
A 48-year-old patient. DM examinations show a cluster of grouped microcalcifications at upper outer left breast (**a**,**b**). At DCE-MRI was observed non-mass enhancement with linear distribution at upper outer quadrant of left breast (**c**).

Based on these premises, our study aims to identify patterns and characteristics in DM that could predict the histologic and molecular subtypes of breast lesions and to determine the concordance of these features with DCE-MRI findings. An illustrative example is presented in Figure 1, where a grouped distribution of fine pleomorphic microcalcifications corresponds to a small non-mass, linear enhancement with a heterogeneous pattern indicative of unifocal ductal carcinoma in situ (DCIS).

Contributions are as follows:

1. Our research offers a novel approach to correlating DM characteristics with histological and molecular subtypes of BC, enhancing the predictive accuracy of imaging methods;
2. We address existing gaps in the literature by exploring the potential imaging correlations between microcalcifications and B3 lesions, expanding the understanding of these lesions’ malignant potential;
3. By examining the utility of DCE-MRI in evaluating microcalcifications, our study contributes to refining diagnostic protocols and surgical planning strategies;
4. Our findings underscore the importance of integrated imaging approaches in the comprehensive assessment of breast lesions, potentially leading to more personalized and effective patient care strategies.

## 2. Materials and Methods

### 2.1. Study Design and Patient Population

Our study utilized a systematically curated database from the Department of Radiological, Oncological, and Pathological Sciences at the Sapienza University of Rome, documenting suspicious microcalcifications detected through DM. We conducted a retrospective analysis of data spanning January 2020 to April 2023.

**Figure 2:**
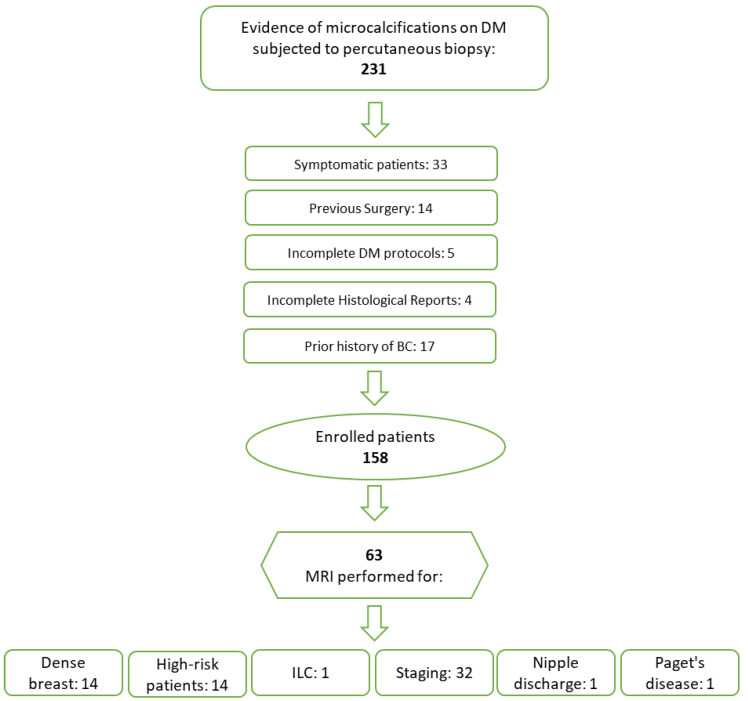
Patient enrollment flowchart.

We selected patients for our study who had routine mammograms showing suspicious microcalcifications and who then had a biopsy at our hospital. Exclusion criteria encompassed patients with a prior history of BC, breast surgery, DM performed due to symptoms, and incomplete DM protocols or histological results (Figure 2).

This monocentric, retrospective study was approved by our Institute’s Ethics Committee, and the need for patient consent was waived.

### 2.2. Digital Mammography and Microcalcifications

Mammograms were conducted using the low-dose Giotto Class system (IMS Giotto, Bologna, Italy).

For each patient, we obtained two standard views: craniocaudal (CC) and oblique mediolateral (MLO). In patients with dense breasts, classified as ACR b or c [3], we also performed additional tomosynthesis in the MLO view to improve the clarity of the breast tissue classification according to the BI-RADS category. Two radiologists (F.P. and V.R.) with 20 and 6 years of experience in breast imaging, respectively, conducted the DM reviews in consensus. Readers were aware of the study’s objective but were unaware of any prior breast examination results and clinical or histopathological information.

The microcalcifications were classified according to the ACR BI-RADS^®^ 5th Edition Atlas [3]. DM with BI-RADS 0, 1, 2, or 6 were excluded from the study.

The morphological features of the microcalcifications were documented, and the size of the lesions was measured by determining the largest diameter. In cases of multifocal, multicentric, or bilateral extension of the disease, the microcalcification region with the largest dimensions (greater diameter) was designated as the index lesion and subjected to statistical analyses.

### 2.3. Breast DCE-MRI

Some enrolled patients underwent breast DCE-MRI before percutaneous biopsy (Figure 2).

All the DCE-MRI examinations were performed using a 3 Tesla machine (Discovery 750; GE Healthcare, Milwaukee, WI, USA).

The DCE-MRI protocol comprised the following:

- Axial two-dimensional (2D) fast spin-echo (FSE) T2-weighted fat-suppressed (FS) sequence based on a three-point Dixon technique (IDEAL);
- Axial dynamic dual-echo 3D spoiled gradient-recalled (DISCO) T1-weighted fat-suppressed sequence, acquired once before and nine times after the injection of contrast media (Gadoteridol-Prohance 279.3 mg/mL; Bracco Imaging Italia S.r.l., Milano, Italy).

Lesions identified in DCE-MRI were categorized based on the presence of contrast enhancement and further classified into non-mass and mass lesions. For non-mass lesions, distribution, and internal pattern enhancement were noted, while mass lesions were characterized by shape, margins, and internal enhancement [3,13]. The presence of peritumoral edema was also documented [14].

In instances of multifocal, multicentric, or bilateral disease, a lesion demonstrating post-contrast enhancement was identified as the index lesion. This designation was based on an assessment of its morphological and spatial characteristics in comparison to those of the DM.

### 2.4. Percutaneous Biopsy and Histopathological Findings

Experienced breast radiologists, each with at least 6 years of expertise, performed ultrasound-guided core needle biopsy (CNB) and stereotactic vacuum-assisted biopsy (VAB). In the presence of non-unifocal disease, the biopsy targeted one or more suspicious areas, always including the index lesion.

For lesions visible on ultrasound, CNB was executed using a 12 MHz linear probe (Toshiba SSA-700A; Tokyo, Japan/Philips Affiniti70G; Amsterdam, The Netherlands) equipped with a 14-gauge semi-automatic biopsy needle (Precisa, Hospital Service S.p.A., Aprilia, Italy). A minimum of four samples were obtained from each lesion.

VAB was conducted under mammographic guidance (Giotto Class, IMS Giotto, Bologna, Italy) using an 11-gauge semi-automatic needle (Mammotome; Ethicon Endo-surgery, Cincinnati, OH, USA), with at least 12 samples collected from each lesion.

All samples were evaluated by expert pathologists, each with a minimum of 10 years of experience. The analysis was categorized using the NHS B-code system [15].

Benign lesions were classified under Category B2.

Category B3 [7], in descending risk order, includes atypical ductal hyperplasia (ADH), lobular neoplasia (LIN), which covers both lobular carcinoma in situ (LCIS) and atypical lobular hyperplasia (ALH), radial sclerosing lesions (RSL), papillary lesions (PL), flat epithelial atypia (FEA), fibroepithelial lesions, and others [16].

No cases of B4 lesions occurred in our patient cohort.

Category B5 denotes unequivocally malignant lesions, subdivided into B5a (in situ) and B5b (invasive) [5].

For all malignant lesions, the molecular subtype was determined based on immunohistochemical characteristics. Tumors were classified as luminal A-like, luminal B-like HER2-negative, luminal B-like HER2-positive, HER2-positive, and triple-negative (TN), adhering to the classifications set forth by the St. Gallen Consensus Conference [17].

### 2.5. Statistical Analysis

Statistical analyses were conducted using IBM SPSS Statistics v.28 (Chicago, IL, USA), with significance set at *p*-values < 0.05. The normality of continuous variables’ distribution was evaluated using the Kolmogorov–Smirnov Z-test, and these variables were presented as median and range. For categorical variables, the χ^2^ test was utilized for comparisons, incorporating the Bonferroni correction for post hoc analysis.

To determine the predictive value of imaging-derived features for the various molecular subtypes of BC, both univariate and multivariate logistic regression analyses were performed. Variables that achieved a *p*-value < 0.05 in the univariate analysis were subsequently included in the multivariate analysis.

The histopathological findings from biopsy specimens served as the ultimate reference standard.

## 3. Results

### 3.1. Study Population

A total of 158 patients were enrolled in the study. The Kolmogorov–Smirnov analysis revealed non-normal distributions for both age and lesion size (Table 1).

Regarding biopsy procedures, 63 patients underwent CNB, and 95 underwent VAB.

Lesions characterized by CNB included 32 cases with a mammographic opacity and 31 cases with only microcalcifications evident on DM.

VAB biopsied 33 lesions with mammographic opacities and 62 lesions without, showing a significant difference in the distribution of mammographic opacities between the two biopsy methods (*p* = 0.045).

Out of the evaluated lesions, 55 were benign, and 103 were classified as malignant (84) or B3 (19).

For detailed insights into the histological and molecular subtypes, refer to Table 2 and Table 3.

Spearman’s correlation analysis showed a significant positive correlation between age and the presence of malignant lesions (ρ = 0.223; *p* = 0.005). Additionally, the χ^2^ test revealed an association between menopausal status and the occurrence of malignant lesions (*p* = 0.006). However, within the group of malignant lesions, no significant associations were found between menopausal status and either histological subtype (*p* = 0.456) or molecular subtype (*p* = 0.405).

### 3.2. Digital Mammography

In our cohort, we identified 127 cases of unifocal, 18 cases of multifocal, 9 cases of multicentric, and 4 cases of bilateral microcalcifications. Microcalcifications were associated with an opacity in 65 cases. The median lesion size on DM is 15 mm (3–109 mm), with 92 lesions < 20 mm and 66 lesions ≥ 20 mm.

Spearman’s correlation analysis revealed an inverse correlation between age and lesion size (ρ = −0.23; *p* = 0.004).

No significant correlation was observed between the presence of opacities and the occurrence of malignant lesions (*p* = 0.429). Similarly, no significant associations were identified with the histological subtype (*p* = 0.03, which was not significant after Bonferroni correction) or molecular subtype in malignant lesions (*p* = 0.773).

The extent of disease also showed no significant association with malignant outcomes (*p* = 0.155), histological subtype (*p* = 0.419), or molecular subtypes (*p* = 0.327).

The features of microcalcifications on DM are described in Table 4.

The χ^2^ analysis conducted on the overall dataset revealed a correlation between the morphology of microcalcifications and the occurrence of malignant lesions (*p* = 0.005). After applying the Bonferroni correction, a distinct association was highlighted between fine linear or fine linear branching microcalcifications and the malignancy of lesions (*p* < 0.001). However, the analyses performed on the histological subtypes (*p* = 0.436) and molecular subtypes (*p* = 0.006, with no significance detected after Bonferroni correction) of malignant lesions did not reveal a direct correlation.

**Figure 3:**
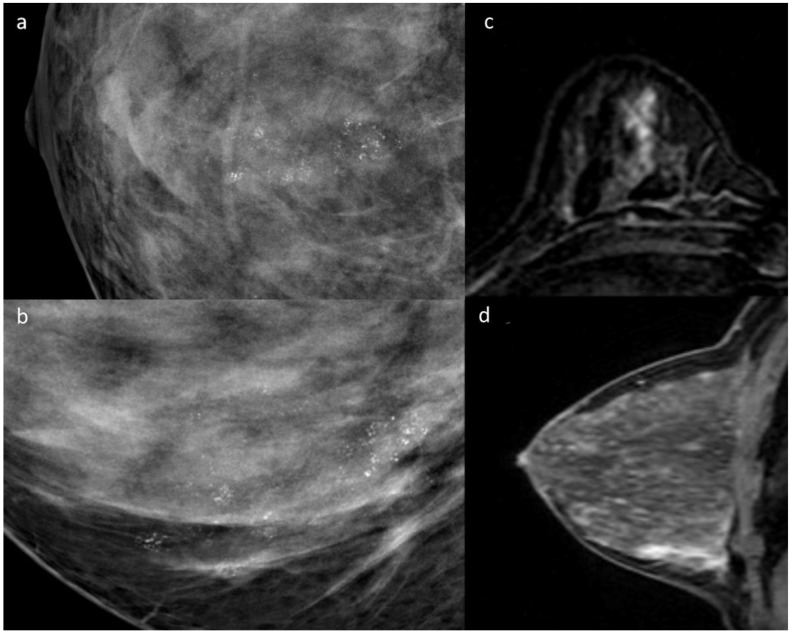
A 35-year-old female with biopsy result B3—ADH. Mammography shows the presence of amorphous microcalcifications with regional distributions at the union of the lower quadrants (**a**,**b**). DCE-MRI shows linear heterogeneous non-mass enhancement (**c**,**d**).

The analysis of B3 lesion subtypes indicated a robust relationship between the variables (*p* < 0.001). Specifically, after the Bonferroni correction, a significant correlation between the amorphous microcalcifications and ADH (*p* = 0.013) (Figure 3), as well as between the coarse heterogeneous (*p* < 0.0001) and fine pleomorphic (*p* = 0.008) morphologies and ALH, was observed. Furthermore, a significant association was identified between FEA and fine pleomorphic morphology (*p* = 0.009).

In examining the relationship between breast lesion subtypes and the distribution of microcalcifications, noteworthy correlations emerged. These included associations between B3 lesions and grouped distribution (*p* < 0.001), B2 lesions and regional distribution (*p* = 0.002), and malignant lesions with both linear (*p* = 0.001) and segmental (*p* = 0.001) distributions.

When evaluating the histological subtypes of malignant lesions (*p* = 0.367) and B3 lesions (*p* = 0.399), no evident correlation was found. However, an association was observed among the molecular subtypes of malignant lesions (*p* = 0.005). After applying the Bonferroni correction, a correlation was noted between the TN subtype and segmental distribution (*p* < 0.001). It is crucial to contextualize this latter result, considering that only two TN lesions with segmental morphology were included in the data, and both were identified as DCIS.

### 3.3. Breast DCE-MRI

Out of 158 patients who underwent DM, 63 patients proceeded to have DCE-MRI.

A total of 58 index lesions showed post-contrast enhancement, comprising 11 B2 lesions, 6 B3 lesions, and 41 malignant lesions.

The median size was 22.5 mm (7–125 mm), with 23 lesions < 20 mm and 35 ≥ 20 mm.

A total of 14 enhanced lesions were identified as mass lesions and 44 as non-mass lesions.

Concerning non-mass lesions, 14 exhibited a linear distribution, 13 a segmental distribution, and 17 a regional distribution. Enhancement patterns varied, with 7 lesions characterized by homogeneous enhancement, 31 by heterogeneous enhancement, and 6 by clumped enhancement.

For mass lesions, five presented an oval shape, eight were round, and one was irregular in shape. Eight lesions had circumscribed margins, whereas six featured irregular margins. The enhancement patterns among mass lesions included seven with homogeneous enhancement, six with heterogeneous enhancement, and one with rim enhancement.

Perilesional edema was absent in 59 lesions, while 4 lesions exhibited perilesional edema (Table 5).

A correlation between malignant outcomes and lesion size was observed (χ^2^ *p* = 0.012). However, no correlation was found between tumor size, histological subtypes, and molecular subtypes.

An association was noted between B2 lesions and the absence of post-contrast enhancement (*p* = 0.002), as well as between the presence of enhancement and malignant lesions (*p* = 0.001).

Regarding non-mass lesions, 22 were identified as DCIS, 9 as IDC, 1 as ILC, and 1 corresponding to Paget’s disease. For mass lesions, three were identified as DCIS and five as IDC. Despite the prevalence of non-mass lesions in DCIS cases, no statistical significance was found (χ^2^ *p* = 0.293).

No correlation was observed between the type of enhancement and molecular subtype (*p* = 0.051), and the Bonferroni test confirmed the lack of statistical significance for all variables. Further details are available in Table 6.

**Figure 4:**
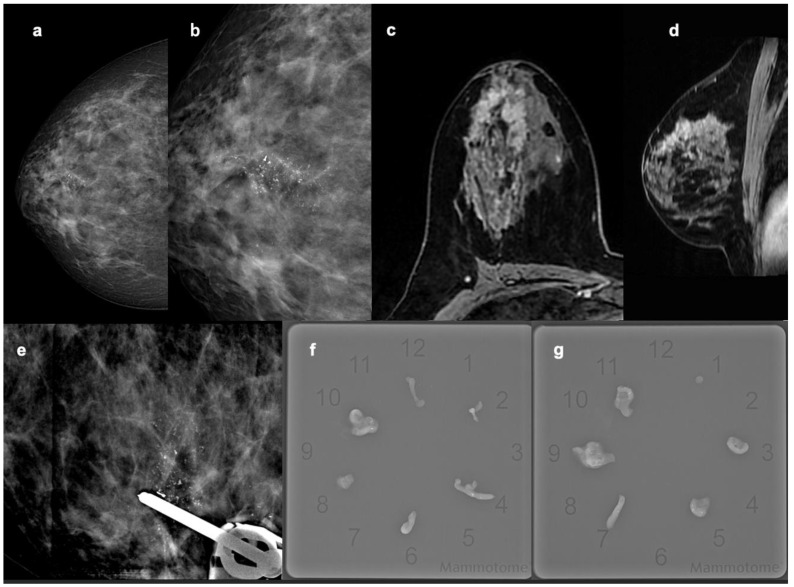
A 48-year-old female with invasive ductal carcinoma, molecular subtype luminal B Her 2+. Mammography in the CC projections shows the presence of regionally distributed amorphous microcalcifications between the upper quadrants of the right breast (**a**,**b**). MRI of the same patient showed a heterogeneous non-mass enhancement with regional distribution in the post-contrast T1w sequences on the axial and sagittal planes (**c**,**d**). Biopsy under stereotaxic guidance of the microcalcifications and radiographic control of micro-histological specimens (**e**–**g**).

The distribution of non-mass lesions was found to be associated with histological subtypes (*p* = 0.004), particularly in IDC lesions, which more frequently exhibit regional distribution (*p* < 0.001) (Figure 4). No association is observed between molecular subtypes and the distribution of non-mass lesions (*p* = 0.266).

### 3.4. DM vs. Breast DCE-MRI

The majority of microcalcification clusters not associated with an opacity (39 lesions) on mammography demonstrated a non-mass morphology on DCE-MRI (30/39), yet this finding did not achieve statistical significance (*p* = 0.064).

No correlation was detected between the type of enhancement in DCE-MRI and the morphology (*p* = 0.414), distribution (*p* = 0.14), and size (*p* = 0.755) of the microcalcification clusters.

The absence of peritumoral edema, observed in 59 lesions, was correlated with the absence of opacity in 39 of these cases (*p* = 0.008). Conversely, the presence of fine linear branching microcalcifications was strongly linked to the occurrence of edema, as evidenced in all four observed cases (*p* < 0.001).

### 3.5. Univariate and Multivariate Logistic Regression

The results of the regression analysis are summarized in Table 7.

## 4. Discussion

Microcalcifications are pivotal in detecting BC at its early stages [18]. They are the sole indicator for approximately half of the non-palpable BC cases discovered through mammography [19] and are instrumental in identifying up to 90% of ductal carcinomas in situ [20].

Our research found a notable link between the age and menopausal status of patients and the incidence of malignant lesions. The increased occurrence of microcalcifications in older women might be attributed to the natural transition toward fatty breast tissue, which is part of the mammary gland’s involution starting in the fourth decade of life. Beyond the age of 60, dense nodular breast tissue becomes infrequent, possibly due to hormonal changes, including the effects of hormone replacement therapy, which can cause a widespread increase in mammographic density [21]. Additionally, women above 50 are more likely to participate in mammography screening programs. According to the European Breast Guidelines, organized screening is recommended for women aged 40 to 75 who are at average BC risk, focusing on DM and, when applicable, the US [22]. Regular DM screenings are proven to be the most effective way to catch the disease in its early stages and reduce BC mortality rates [22,23].

In our study, 41% of microcalcifications were associated with opacity, and the median lesion size on DM was 15 mm, with an inverse correlation between age and lesion size. These data could be explained because most women under 50 years of age had heterogeneous or dense breasts on DM, which often hampered the detection of opacities but not microcalcification. Another explanation could be that microcalcifications were positively associated with the presence of in situ lesions or tumors with an intraductal component, as already shown by others [24], and these histological types were themselves more frequent in younger women [19].

One of the study’s purposes was to clarify whether diagnostic DM could be a reliable noninvasive predictor of histological tumor type and molecular subtype of BC. Out of most of the patients with BC included in this study, 54.7% were affected by DCIS, 3.5% by ILC, and about 40% by invasive carcinoma NST. Our distribution substantially reflected data reported in the literature [19,25].

In this regard, we found a specific association between fine-linear or fine linear branching morphology of microcalcifications and the malignancy of the lesions. According to Kim et al., the positive predictive values (PPVs) for microcalcifications with suspicious morphology in their study were 7.9% for amorphous, 17.8% for coarse heterogeneous, 63.2% for fine pleomorphic, and 100% for fine linear/fine linear branching [26]. Our results can be related to several studies showing decreased survival associated with fine linear branching morphology of calcification among women diagnosed with breast cancer [27,28,29,30].

Despite the well-documented link between microcalcifications and BC in the existing literature [5,6], there appears to be a gap in imaging studies specifically correlating microcalcifications with B3 lesions. B3 lesions, as defined by European and United Kingdom breast pathology guidelines, constitute a diverse group with a wide spectrum of potential malignancy [7,8,9,10,11,12], with ADH and LN presenting the highest risk. The scarcity of research focusing on the appearance of B3 lesions on DM prompted our study to explore whether these lesions have distinct microcalcification morphologies and patterns. Our findings indicate a significant correlation between amorphous microcalcifications and ADH, as well as between coarse heterogeneous and fine pleomorphic microcalcifications and ALH. Additionally, a significant link was found between FEA and fine pleomorphic microcalcifications. These insights enhance our ability to differentiate potentially borderline microcalcifications from benign ones. This differentiation is particularly crucial when considering the discordance often observed between radiological assessments (classified as BI-RADS 4b or 4c) and histopathological diagnoses of B3, a discrepancy that previous studies have identified as a significant predictor for lesion upgrade [31,32,33]. Currently, each B3 case requires evaluation by a multidisciplinary team, reflecting the complexity and uniqueness of each patient’s situation, without a one-size-fits-all approach [32]. Our analysis identified significant correlations between the linear and segmental distribution patterns of malignant lesions, aligning with findings reported in the existing literature [27,34]. Specifically, Kim et al. noted that fine pleomorphic calcifications with linear and segmental distributions have a high predictive value for malignancy (PPV of 93.8%) and should invariably be classified as BI-RADS 4c [27].

Additionally, our study found a notable correlation between the grouped distribution of B3 lesions. This was especially true for FEA and ALH, where 100% of the cases presented as grouped microcalcifications. According to Mariscotti et al. [35], such lesions have the potential to be upgraded to malignancy. However, it is important to note that this inference is speculative at this stage, given the limited size of our B3 lesion sample. To validate these findings, a statistical analysis with a larger cohort of B3 lesions would be necessary.

While certain studies in the literature indicate a potential link between the histological subtype of breast cancer and the distribution and morphology of macrocalcifications, our research did not find a significant correlation. Despite the common diagnosis of DCIS on DM, characterized by fine linear microcalcification distribution [36], our results did not support a significant correlation. Similarly, Kim et al., in their examination of 94 DCIS cases detected through screening mammography, reported no significant correlation between the morphology and distribution of microcalcifications and receptor subtypes [37].

In a different study, Bae et al. analyzed 101 DCIS cases with microcalcifications and observed that fine pleomorphic and fine linear branching microcalcifications with segmental distribution were mainly seen in HER2+ DCIS cases, whereas punctate and amorphous microcalcifications with grouped distribution were more prevalent in ER/PR-positive cases [38]. Contrary to these findings, our study identified an association between molecular subtypes of malignant lesions and distribution patterns, specifically noting a correlation between the TN subtype and segmental distribution. TN breast cancers (TNBCs) are recognized for their biological and clinical aggressiveness and can exhibit imaging features that sometimes resemble benign lesions, such as opacity [23]. This discrepancy with the existing literature could be explained by the fact that the TN cases in our study were DCIS.

The second aim of our study was to investigate the relationship between microcalcifications detected in DM and characteristics seen in DCE-MRI. Currently, there is insufficient information on the diagnostic effectiveness of MRI for patients presenting with new or enlarging microcalcifications. When an MRI identifies a positive lesion that corresponds to an area with new or expanding microcalcifications observed in mammography, a biopsy is typically advised. Conversely, patients with unchanged microcalcifications may proceed with regular mammography follow-ups, irrespective of the MRI findings [39,40,41].

In our study, 39.9% of patients with suspicious microcalcifications detected on DM underwent DCE-MRI. The relatively small proportion of patients receiving DCE-MRI compared to those assessed with DM represents a limitation of our research. Despite the limited sample size, our findings are consistent with those reported in the literature. The high prevalence of ductal carcinoma in situ (DCIS), which constitutes 61% of the malignant lesions identified, is recognized as a factor that may reduce the specificity of DCE-MRI in cases involving microcalcifications. According to a review by Bennani-Baiti et al. [42], out of 1843 lesions examined, 106 were falsely negative (5.8%), with 68 (64.2%) of these being exclusively DCIS. The most frequent manifestation of microcalcifications was non-mass lesion enhancement, accounting for 69.8% of cases, aligning with the literature that reports a prevalence of 58.4% [43,44]. In particular, MRI features of DCIS are related to specific growth patterns within the ducts and typical neovascularization: tumor cells can directly release angiogenic factors, resulting in a rim of microvessels adjacent to the basement membrane of affected ducts, or indirectly via recruitment of accessory cells, leading to diffuse stromal vascularity [45].

Perilesional edema was observed in only four lesions, all of which were malignant, specifically IDC. Perilesional edema, visible as pathological hyperintensity on MR T2-weighted images surrounding a tumor, is thought to result from proteolysis and neoangiogenesis associated with tumor growth and progression, leading to inflammatory cytokine release and increased vascular permeability, which causes fluid transudation into the surrounding extracellular space [14,46].

In accordance with our previous research [14,47], Costantini et al. [48] also found that peritumoral edema in DCE-MRI is linked to aggressive TNBCs. Other studies have underscored the negative prognostic impact of peritumoral edema [49,50,51]. Our current analysis revealed a significant association between the presence of peritumoral edema and fine linear branching microcalcification on DM, suggesting that the identification of such microcalcification should be considered a prognostic marker indicative of malignancy and overall poorer prognosis. This insight could enhance the assessment of breast cancer patients.

DCE-MRI has the potential to reduce unnecessary breast biopsies, especially in cases of microcalcifications with a low risk of malignancy. Furthermore, DCE-MRI can aid in surgical planning after a percutaneous biopsy confirms a malignancy, helping to evaluate factors like lesion size, multifocality, multicentricity, and bilaterality for patients eligible for breast-conserving surgery [43,44,52,53].

Our study is subject to limitations, including its retrospective nature and the small size of the patient cohort for the groups of TN and HER2+ BC. The evaluation of DM and DCE-MRI datasets by only two readers without considering interobserver variability and the uneven distribution of cases across subgroups may affect the reliability of our findings. Future research with larger patient cohorts is needed for more definitive conclusions on this topic.

## 5. Conclusions

Our study underscores the importance of adapting screening protocols based on patient age and menopausal status. Such adjustments could significantly enhance the early detection of malignant microcalcifications, which are more prevalent in older or menopausal women. Additionally, there is a crucial need for specialized training for radiologists. Recognizing specific microcalcification morphologies, particularly fine linear and branching patterns, is vital as these are strongly correlated with malignancy and can influence diagnostic accuracy. Furthermore, the integration of DM with DCE-MRI provides a more comprehensive evaluation of breast lesions, particularly in ambiguous or complex cases. This combined approach can help in accurately identifying and characterizing breast lesions, potentially leading to more precise diagnoses and tailored treatment strategies. In instances where imaging results suggest a high probability of malignancy, particularly with microcalcifications of suspicious morphology, our study supports the use of VAB. This method not only confirms the diagnosis but also aids in the planning of appropriate therapeutic interventions.

In conclusion, our study provides a comprehensive evaluation of microcalcifications assessed on DM and their relationships with histology and breast DCE-MRI findings. 

## Figures and Tables

**Table 1:** Overview of the breast cancer patient cohort.

Patient Cohort Features	*N*	Median	%
Patients	158		
Age	29–89 years (min.–max.)	50 years	
Menopausal stage	81		51.3%
Pre-menopausal stage	77		48.7%
Tumor size on DM	3–109 cm (min.–max.)	15 cm	

**Table 2:** Histological subtypes.

B2 Lesions		B3 Lesions		Malignant Lesions	
Fibrocystic mastopathy	25	FEA	9	DCIS	46
Fibroadenoma	6	Radial scar	1	IDC	34
Micropapillary apocrine metaplasia	4	ALH	4	ILC	3
Steatonecrosis	1	ADH	5	Paget disease	1
Sclerosing adenosis	7				
Fibroadipose involution	4				
Columnar cell alteration	5				
Typical ductal hyperplasia	1				
Stromal fibrosis	1				
PASH	1				
Total	55	Total	19	Total	84

**Table 3:** Molecular and histological subtypes.

		Histological Subtype				Total
		DCIS	IDC	ILC	Paget Disease	
Molecular subtype	Luminal A	19	8	3	0	30
	Luminal B Her−	12	12	0	1	25
	Luminal B Her+	5	10	0	0	15
	Her2+	8	4	0	0	12
	TN	2	0	0	0	2
Total		46	34	3	1	84

**Table 4:** The distributions and morphology of microcalcifications were assessed through DM.

DM Microcalcifications	*N*	%
Distribution		
Grouped	83	52.5%
Diffuse	5	3.2%
Regional	45	28.5%
Linear	11	6.9%
Segmental	14	8.9%
Morphology		
Amorphous	48	30.4%
Coarse heterogeneous	39	24.7%
Fine pleomorphic	52	32.9%
Fine linear or fine linear branching	19	12.0%

**Table 5:** Peritumoral edema according to histological subtype.

	Peritumoral Edema	
Histological Subtype	Presence	Absence
DCIS	0	25
IDC	4	10
ILC	0	1
Paget disease	0	1
Non-malignant lesion	0	22
Total	4	59

**Table 6:** Molecular subtypes and post-contrast enhancement type.

	Molecular Subtype				
	Luminal A	Luminal B Her−	Luminal B Her+	Her2+	TN
Mass	0	5	2	1	0
Non-mass	15	7	9	2	0

**Table 7:** Predictors of B2, B3, and B5 lesions. Statistically significant results are in bold.

	Univariate Analysis		Multivariate Analysis (Stepwise Method)	
	OR (CI 95%)	*p*-Value	OR (CI 95%)	*p*-Value
Age	1.040 (1.012–1.069)	**0.005**	1.052 (1.021–1.083)	**<0.001**
Menopause	0.356 (0.187–0.679)	**0.002**	Eliminated *	
Microcalcifications distribution	1.534 (1.191–1.975)	**<0.001**	1.693 (1.285–2.231)	**<0.001**
Microcalcifications morphology	1.301 (0.953–1.776)	0.098		
Associate opacity	1.293 (0.684–2.446)	0.429		
Enhancement lesion	Out of scale	0.999		
Non-mass distribution	1.952 (0.826–4.614)	0.128		

OR = odds ratio, CI = confidence interval. * The variable was eliminated in the first step of the analysis as it was not significant when evaluated in the multivariate model.

## Data Availability

Data available on request.
